# Case Report: Infantile hypercalcemia type 1 due to CYP24A1 pathogenic variants—three adult patients with nephrolithiasis and nephrocalcinosis successfully treated with fluconazole

**DOI:** 10.3389/fmed.2026.1925619

**Published:** 2026-09-01

**Authors:** Nikolina Vucenovic Basic, Bodo Beck, Annemarie Balasko, Ivana Vukovic Brinar, Margareta Fistrek Prlic

**Affiliations:** 1 Department of Internal Medicine, General Hospital Sibenik, Sibenik, Croatia; 2 Department of Internal Medicine, University Hospital Centre Zagreb, Zagreb, Croatia; 3 Institute of Human Genetics, University Hospital Cologne, Cologne, Germany; 4 School of Medicine, University of Zagreb, Zagreb, Croatia

**Keywords:** fluconazole, infantile hypercalcemia, nephrocalcinosis, nephrolithiasis, vitamin D

## Abstract

Infantile hypercalcemia type 1 (HCINF1) is a rare genetic disorder caused by pathogenic variants in the CYP24A1 gene, which encodes the vitamin D–catabolic enzyme 24-hydroxylase responsible for inactivating 1,25-dihydroxyvitamin D₃. The clinical presentation ranges from severe forms diagnosed in infancy—characterized by hypercalcemia, dehydration, and vomiting—to milder forms that are often identified in adulthood during evaluation for recurrent nephrolithiasis. We present two adult female patients with late-onset HCINF1, both initially referred to a nephrologist for nephrolithiasis and nephrocalcinosis, and one male patient with an infantile presentation who began fluconazole therapy in adulthood. This case series highlights rare late-onset manifestations of HCINF1 associated with recurrent nephrolithiasis, secondary distal renal tubular acidosis (dRTA), and variable degrees of renal function impairment. It emphasizes the importance of considering genetic testing in adults with unexplained hypercalcemia and nephrolithiasis and supports the therapeutic role of fluconazole in selected cases.

## Introduction

Nephrocalcinosis (NC) is characterized by an abnormal accumulation of calcium in the renal parenchyma (1, 2). It is closely associated with kidney stone formation, i.e., nephrolithiasis (NL), and both conditions are associated with significant morbidity, including an increased risk of renal failure and chronic kidney disease (CKD) (3, 4). Genetic and environmental factors contribute to the development of both NL and NC. Although uncommon in the general population, monogenic causes of NL and NC account for a substantial proportion of the disease burden (5, 6). Genotypes affecting many enzyme pathways, such as hepatic glyoxylate metabolism and vitamin D, are associated with both NL and NC, and different phenotypes have been attributed to the monogenic causes of NL and NC (5). While causal or supportive therapies may be limited, the successful treatment of some monogenic forms of NL and NC has been reported (7). Therefore, determining the genetic cause of kidney diseases, including NL and NC, can guide treatment and provide important prognostic information.

Regarding vitamin D metabolism, the second stage of vitamin D activation, which results in the production of the active metabolite 1,25-dihydroxyvitamin D3, occurs in the kidneys. Pathogenic variants in CYP24A1, the gene encoding 1,25-hydroxyvitamin-D3-24-hydroxylase, underlie the inability to catabolize this active vitamin D metabolite (8). A small cohort of infants with this genetic cause of disease experienced serious side effects from the public health practice of adding vitamin D to formula milk, including nephrocalcinosis and hypercalcemia, which resulted in the identification of CYP24A1 variants and the subsequent recognition of HCINF1 (9, 10).

Further research has shown that pathogenic variants in the CYP24A1 gene result in two distinct phenotypes, both of which frequently involve nephrocalcinosis (10). Regarding childhood manifestations, in addition to hypercalcemia, infants with HCINF1 often present with severe dehydration, vomiting, and failure to thrive (10). Nephrocalcinosis is often apparent on ultrasound at the time of diagnosis (10). Eliminating exogenous sources of vitamin D (mostly supplements), fluid resuscitation, dietary modifications, and avoiding further UV light exposure are the major goals of treatment for these patients. It has also been demonstrated that adults with nephrolithiasis, hypercalciuria, or incidentally detected nephrocalcinosis may exhibit a second, late-onset phenotype of HCINF1. Usually milder in nature, this phenotype is not always associated with a history of past vitamin D administration (11).

Antifungal medications, such as fluconazole and ketoconazole, are non-specific P450 enzyme inhibitors that block 1α-hydroxylase encoded by CYP27B1, the enzyme responsible for converting 25-hydroxyvitamin D into biologically active 1,25-dihydroxyvitamin D. This effect has been shown to help reduce the production of the active form of vitamin D. By suppressing the activity of CYP27B1, azoles can effectively reduce circulating calcitriol concentrations and thereby diminish intestinal calcium absorption. In the context of CYP24A1 deficiency, in which impaired catabolism leads to the pathological accumulation of calcitriol, this upstream inhibition provides a functional counterbalance that can reduce hypercalcemia and hypercalciuria. Consequently, azole therapy may represent a targeted metabolic intervention that may stabilize biochemical abnormalities and mitigate renal complications in patients with HCINF1 (12–14). Tebben et al. reported the case of an adult man with a splice-site mutation in CYP24A1 who was treated with ketoconazole (200 mg three times daily). A significant reduction in urinary calcium excretion and a modest reduction in serum calcium were achieved after 2 months of treatment (12). Nesterova et al. described the treatment of a patient with a *CYP24A1* mutation using ketoconazole (up to 800 mg/day), resulting in reductions in serum calcium, urine calcium, and 1,25-dihydroxy vitamin D levels (13). Regarding treatment with fluconazole, Sayers et al. described the case of an adult patient with CYP24A1-associated hypercalcemia. Successful control of serum calcium levels and reduced urinary calcium excretion were accomplished using a low dose of fluconazole (50 mg daily) (14). The same low-dose regimen of fluconazole was used to treat our patients.

However, long-term treatment with antifungal medications might not be preferred due to hepatotoxicity, which is a common side effect of these medications. It has recently been demonstrated that rifampicin can effectively reduce 1,25-dihydroxyvitamin D3 levels and improve hypercalcemia in patients with pathogenic variants in the *CYP24A1* gene (8, 14, 15).

## Case series

### Case 1

**Figure 1:**
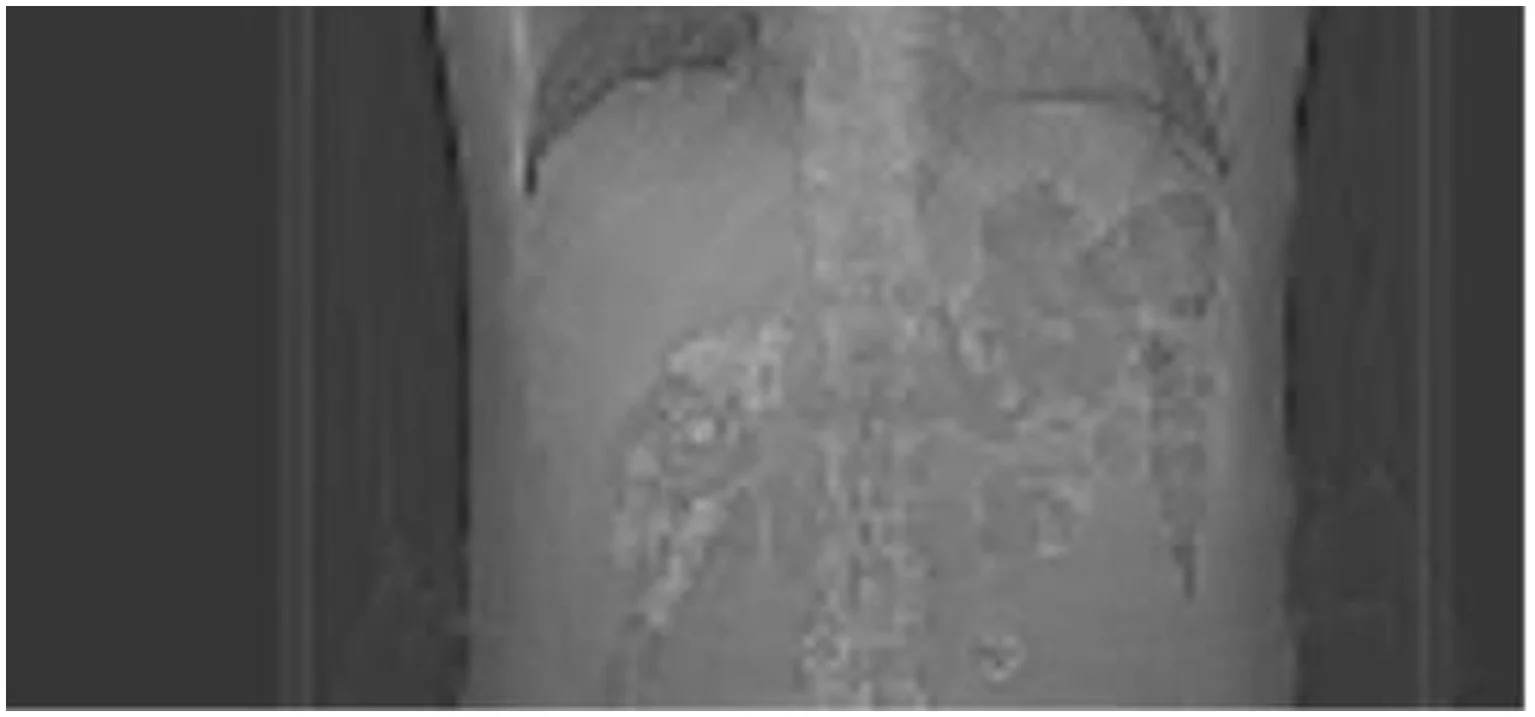
The low-dose non contrast CT scan of upper abdomen region showing sever bilateral nephrocalcinosis (Patient 1).

A 46-year-old female patient was referred to a nephrologist because of recurrent nephrolithiasis. She had a history of nephrolithiasis and occasional renal colic since adolescence. Additional imaging revealed severe nephrocalcinosis (Figure 1), while laboratory evaluation revealed reduced renal function (eGFR 35 mL/min/1.73 m^2^), hypercalcemia (up to 2.74 mmol/L), hypercalciuria, and an inappropriately suppressed parathyroid hormone (PTH) level (2.26 pmol/L). Parathyroid imaging—including neck ultrasound, sestamibi scintigraphy, and choline PET-CT—showed no evidence of parathyroid adenoma or hyperplasia. The minimally elevated PTH was therefore interpreted as secondary and appropriate for the degree of chronic kidney impairment rather than as evidence of primary hyperparathyroidism.

Laboratory findings also demonstrated borderline hypokalemia, hypocitraturia, and persistently high urine pH levels with normal serum bicarbonate levels, which were consistent with incomplete distal renal tubular acidosis (dRTA). Urinary stone analysis confirmed a predominantly calcium-phosphate composition, which correlates with elevated urinary pH levels.

Genetic analysis was performed using a targeted next gen-sequencing (NGS) assay covering key genes implicated in hereditary stone disease (*ADCY10, AGXT, ALPL, APRT, ATP6V0A4, ATP6V1B1, ATP7B, BSND, CA2, CASR, CLCN5, CLCNKA, CLCNKB, CLDN16, CLDN19, CLPB, CYP24A1, FAM20A, FOXI1, GNA11, GPHN, GRHPR, HNF4A, HOGA1, HPRT1, KCNJ1, MAGED2, MOCOS, MOCS1, MOCS2, OCRL, PRPS1, SLC12A1, SLC22A12, SLC26A SLC2A9, SLC34A1, SLC34A3, SLC3A1, SLC4A1, SLC7A9, SLC9A3R1, UMOD, VDR, XDH*), which identified two pathogenic variants in the *CYP24A1* gene—p.(Leu148Pro) and p.(Leu409Ser), confirming autosomal recessive infantile hypercalcemia type 1 as the underlying cause of longstanding hypercalcemia, hypercalciuria, and nephrocalcinosis. No other monogenic cause of nephrolithiasis or primary dRTA was found, further supporting the interpretation that the tubular acidification defect was a secondary process. The patient’s parents were not eligible for genetic testing, whereas the patient’s brother was heterozygous for the CYP24A1 p.(Leu409Ser) variant, supporting segregation of the familial allele and consistent with autosomal recessive inheritance of HCINF1. The patient was advised to avoid vitamin D supplement intake, as well as sun exposure, and to increase hydration. Given the known role of CYP24A1 loss-of-function in the impaired degradation of active vitamin D metabolites, the patient was started on fluconazole at a dose of 50 mg daily, based on recommendations from previous studies demonstrating that low-dose azole therapy can effectively reduce calcitriol synthesis (14). One month after treatment initiation, both serum calcium concentrations and 24-h urinary calcium excretion normalized, with these effects remaining stable over an 18-month period. There were no significant side effects from the therapy, and liver function remained normal. Kidney function remained stable.

### Case 2

The second patient, a 49-year-old woman, was referred for further evaluation of nephrocalcinosis and recurrent nephrolithiasis. Her medical history was notable for multiple episodes of obstructive nephrolithiasis requiring lithotripsy and JJ stent placement. A positive family history of nephrolithiasis was also reported. Laboratory evaluation revealed hypercalcemia (2.89 mmol/L), marked hypercalciuria, suppressed PTH levels, slightly elevated 25-hydroxyvitamin D levels, and biochemical features suggestive of incomplete distal renal tubular acidosis (dRTA), similar to those observed in the first case. Concomitant hypercalcemia and suppressed PTH levels strongly imply that the hypecalcemia is mediated by a PTH-independent mechanism.

Genetic analysis was performed using a targeted NGS assay (*ADCY10, AGXT, ALPL, APRT, ATP6V0A4, ATP6V1B1, ATP7B, BSND, CA2, CASR, CLCN5, CLCNKA, CLCNKB, CLDN16, CLDN19, CLPB, CYP24A1, FAM20A, FOXI1, GNA11, GPHN, GRHPR, HNF4A, HOGA1, HPRT1, KCNJ1, MAGED2, MOCOS, MOCS1, MOCS2, OCRL, PRPS1, SLC12A1, SLC22A12, SLC26A SLC2A9, SLC34A1, SLC34A3, SLC3A1, SLC4A1, SLC7A9, SLC9A3R1, UMOD, VDR, XDH*). Two pathogenic variants were identified in the *CYP24A1* gene—p.(Arg396Trp) and p.(Leu148Pro), confirming autosomal recessive infantile hypercalcemia type 1 as the underlying etiology. No other monogenic cause of nephrolithiasis or primary dRTA was detected. The patient’s family members were not eligible for genetic testing, precluding the formal segregation analysis of the identified variants.

The patient was advised to avoid vitamin D supplementation and sun exposure and to increase hydration. Treatment with low-dose fluconazole (50 mg) was initiated, resulting in a favorable biochemical response characterized by normalization of serum calcium concentrations and a marked reduction in urinary calcium excretion. The patient reported increased hair loss as a potential side effect of fluconazole. The liver function remained stable.

### Case 3

The third patient was a man who was first hospitalized at 5 months of age due to severe hypercalcemia (serum calcium 5.98 mmol/L), failure to thrive, and osteopenia. Throughout early childhood, he required multiple hospital admissions for nephrolithiasis and ureterolithiasis.

Biochemical evaluation consistently demonstrated hypercalcemia, hypophosphatemia, markedly elevated 25-hydroxyvitamin D (157 nmol/L) with inappropriately normal-to-elevated 1,25-dihydroxyvitamin D (78 pmol/L), elevated alkaline phosphatase, and reduced urinary citrate accompanied by elevated urinary oxalate excretion. He also exhibited pronounced hypercalciuria (13.3 mmol/24 h urine). Renal ultrasound repeatedly confirmed persistent nephrolithiasis, and bone densitometry demonstrated osteopenia.

At 8 years of age, because of symptomatic obstructive calculi, the patient underwent left-sided extracorporeal shock wave lithotripsy (ESWL). Subsequent genetic testing, performed in the same way as in the first two cases, identified a homozygous pathogenic missense variant in the *CYP24A1* gene (p. Arg396Trp), establishing the diagnosis of HCINF1. Both of the patient’s parents were heterozygous carriers of the same CYP24A1 variant, consistent with autosomal recessive inheritance of the disease.

Despite conservative management, including avoidance of vitamin D supplements, excessive sun exposure, and increased hydration, he continued to experience biochemical activity and intermittent hypercalcemic episodes during adolescence. At 18 years of age, fluconazole therapy (50 mg) was initiated as a targeted strategy to reduce excessive vitamin D activation and improve calcium homeostasis. After 4 months of treatment, serum calcium concentrations normalized. The patient did not experience any side effects from the treatment.

The clinical characteristics, as well as the fluconazole dosage and therapeutic effect of all patients, are presented in Table 1.

**Table 1:** Clinical characteristics of the patients.

Clinical characteristics	First patient – Female, 46 years			Second patient – Female, 49 years			Third patient – Male, 18 years		
Family history of nephrolithiasis	Negative			Positive			Negative		
Biochemical parameters	Baseline	1-month follow-up	End of follow-up (18 months)	Baseline	2-month follow-up	End of follow-up (7 months)	Baseline	4-month follow-up	End of follow-up (16 months)
Serum calcium levels (ref. 2.14–2.53 mmoL/L)	2.66	2.54	2.50	2.89	2.61	2.7	2.70	2.66–2.53	2.52
Serum phosphorus levels (ref.0.79–1.42 mmoL/L)	0.88	1.44	N/A	1.4	0.82	0.86	1.10	1.11	1.07
Renal function eGFR (ref. >60 mL/min/1.73 m2)	36	37	36	41	43	63	95	89	90
Parathyroid hormone levels (ref. 1.6–7.2 pmoL/L)	2.26	N/A	N/A	1.46	N/A	N/A	1.35	N/A	3.39
Calciuria (ref. 2.5–7.50 mmol/24 h urine)	7.63	4.6	3.1–2.08	8.1	N/A	5.59	13.3	4.92	N/A
25-OH vitamin D (ref. > 75 nmoL/L)	126	90	90.5	130	N/A	72	157	104	50
1,25-OH vitamin D (ref. 19.9–79.3)	N/A	33.0	21.0	N/A	45.7	N/A	78	N/A	41.3
Alanine Aminotransferase (ref. 10-36U/L)	33	20	23	14	25	29	23	21	25
Aspartate Aminotransferase (ref.11–38 IU/L)	N/A	30	20	20	25	23	18	19	20
Serum potassium mmol/l (ref. 3.9–5.1 mmoL/L)	3.9	4.4	4.2	4.4	4.8	4.7	4.4	5.2	5.0
Urinary pH levels (ref.5.0–9.0)	8.0	6.5	7.0	7.0	7.0	6.5	5.5	6.0	5.5
Serum bicarbonate mmol/l	24.9	24.2	N/A	23.9	24.1	N/A	24.0	23.7	N/A
Urinary citrate (ref. 107–635 mmol/mol)	37.9	N/A	N/A	133	N/A	N/A	57.2	59.0	N/A
Urinary oxalate (ref. 15–32 mmol/mol)	31.7	N/A	N/A	85	N/A	N/A	87.8	N/A	N/A
Nephrolithiasis	Stable during follow-up period, no renal colic.			Previously, the patient underwent two sessions of lithotripsy and ureterorenoscopy with the placement of a JJ stent in the right renal collecting system. Following initiation of fluconazole therapy, the nephrolithiasis remained stable. The JJ stent was subsequently removed, with no occurrence of renal colic.			Stable during follow-up period, and no renal colic.		
Fluconazole dosage	50 mg once daily			50 mg once daily			50 mg once daily		
Adverse effects	No adverse effects			Hair loss			No adverse effects		
Genetic analysis	Two missense variants p.(Leu148Pro) and p.(Leu409Ser) Brother - heterozygous carrier of p.(Leu409Ser) Parents - not segregated - deceased			Two missense variants p.(Arg396Trp) and p.(Leu148Pro) Other family members were not segregated			Homozygous missense variant p.(Arg396Trp) Parents – heterozygous carriers of the same pathogenic variant		

N/A, not applicable.

## Discussion

This case series highlights the marked phenotypic heterogeneity of HCINF1 caused by pathogenic CYP24A1 variants, ranging from severe infantile-onset disease to adult presentations dominated by recurrent nephrolithiasis, nephrocalcinosis, and chronic kidney disease. Our findings further support the use of low-dose fluconazole as a targeted therapeutic strategy to improve calcium homeostasis in patients with impaired vitamin D catabolism (14).

Loss-of-function mutations in CYP24A1 impair the degradation of 1,25-dihydroxyvitamin D, resulting in increased intestinal calcium absorption, hypercalcemia, and hypercalciuria. While the infantile phenotype is often recognized early due to profound metabolic disturbances, adult-onset disease is frequently underdiagnosed and may be misattributed to more common causes of nephrolithiasis (10, 11). In the adult patients presented here, long-standing calcium phosphate stone disease, nephrocalcinosis, and progressive renal impairment were the predominant clinical features, consistent with previously reported late-onset CYP24A1-associated phenotypes (11).

An additional shared feature among our patients was biochemical evidence suggestive of incomplete distal renal tubular acidosis (dRTA), including persistently elevated urinary pH and hypocitraturia in the absence of systemic metabolic acidosis. Importantly, no monogenic cause of primary dRTA was identified, supporting the interpretation that tubular acidification defects were secondary to nephrocalcinosis-related tubular injury. This observation emphasizes the contribution of renal parenchymal damage to disease progression and stone formation in CYP24A1 deficiency.

Management of CYP24A1-associated hypercalcemia remains challenging. Conservative measures—such as avoidance of vitamin D supplementation, dietary calcium restriction, limitation of UV exposure, and adequate hydration—are essential but often insufficient to fully control hypercalcemia and hypercalciuria, particularly in patients with established nephrocalcinosis or chronic kidney disease. Pharmacological inhibition of vitamin D activation therefore represents a rational and pathophysiology-driven therapeutic approach (14–16).

Azole antifungal agents inhibit cytochrome P450–dependent 1α-hydroxylase (CYP27B1), thereby reducing the synthesis of active vitamin D metabolites. Previous reports have demonstrated biochemical improvement with ketoconazole; however, its long-term use is limited by hepatotoxicity and endocrine adverse effects. Fluconazole, a weaker but more selective P450 inhibitor, has emerged as a potentially safer alternative (14). In our patients, low-dose fluconazole therapy was associated with normalization or substantial reduction in serum calcium levels and a marked decrease in urinary calcium excretion, without significant adverse effects. These findings extend previous single-case reports and suggest that even low doses of fluconazole may be sufficient to achieve meaningful biochemical control.

Baseline serum calcium concentrations in our patients were lower than those reported in previously published cases, and normocalcemia was achieved within a 1- to 3-month follow-up period (14).

Rifampicin has recently been proposed as an alternative therapeutic option by inducing CYP3A4-mediated catabolism of active vitamin D metabolites (15). While effective in selected cases, rifampicin therapy is associated with relevant drug–drug interactions and requires careful monitoring. In this context, fluconazole may represent a pragmatic option, particularly in patients requiring long-term therapy or in those for whom rifampicin is contraindicated.

This study has limitations inherent in a small retrospective case series, and long-term safety data for fluconazole in this indication remain limited. Nevertheless, given the rarity of CYP24A1-associated diseases, detailed clinical characterization and treatment response data remain highly informative.

In conclusion, our findings reinforce the importance of considering CYP24A1 variants in patients with PTH-independent hypercalcemia, recurrent nephrolithiasis, or nephrocalcinosis. Low-dose fluconazole appears to be an effective and well-tolerated targeted therapy to reduce hypercalcemia and hypercalciuria across a broad clinical spectrum of CYP24A1-associated disease; however, our experience is limited to a small number of patients. The optimal dosage and duration of treatment with fluconazole for this indication remain uncertain; nevertheless, it may represent a therapeutic option in selected patients, with careful monitoring. Despite the limitations, early genetic diagnosis and individualized management may be critical in preventing irreversible renal damage and improving long-term outcomes.

### Patients’ perspective

The first patient is compliant, has no treatment-related side effects, and has no renal colic. Her kidney function is stable. She leads an active life, which is very important to her. The second patient reported increased hair loss, as a potential side effect of fluconazole, which was not a major concern; however, she remains worried because of her history of multiple urological complications. The third patient has no side effects, no renal colic, and stable kidney function, which allows him to participate in all daily activities.

## Data Availability

Datasets are not publicly available due to privacy laws. Requests to access these datasets should be directed to margareta.fistrek@gmail.com and margareta.fistrek.prlic@kbc-zagreb.hr.
